# High-Dose Chemotherapy in Children with Newly Diagnosed Medulloblastoma

**DOI:** 10.3390/cancers14030837

**Published:** 2022-02-07

**Authors:** Lucie Lafay-Cousin, Christelle Dufour

**Affiliations:** 1 Section of Pediatric Hematology Oncology and Bone Marrow Transplantation, Alberta Children’s Hospital, Calgary, AB T3B 6A8, Canada; 2 Department of Pediatric and Adolescent Oncology, Gustave Roussy, 96805 Villejuif, France; christelle.dufour@gustaveroussy.fr

**Keywords:** newly diagnosed medulloblastoma, childhood, high-dose chemotherapy, trials

## Abstract

**Simple Summary:**

Medulloblastoma is the most common malignant central nervous system tumor in the pediatric population. Treatment modalities are stratified by age, extent of resection, metastatic status, histology, and more recently, tumor biology. Maximal surgical resection followed by risk-adapted craniospinal irradiation and adjuvant chemotherapy have produced the best survival for medulloblastoma. Although survival is, in general, quite high, some caveats and drawbacks argue for continuous adaptations of this treatment strategy. Clearly, for children with metastatic disease, there is no established standard chemotherapy regimen, even if there is a consensus that chemotherapy brings additional benefit. The treatment of infants and very young children is particularly challenging, as irradiation of the developing brain leads to substantial neurocognitive impairment. Some clinical trials have explored the possible efficacy of high-dose chemotherapy in childhood medulloblastoma, and here we have focused on clinical trials for infants and children with newly diagnosed medulloblastoma.

**Abstract:**

High-dose chemotherapy with stem cell rescue has been used as an adjuvant therapy or as salvage therapy to treat pediatric patients with brain tumors, and to avoid deleterious side effects of radiotherapy in infants and very young children. Here, we present the most recent trials using high-dose chemotherapy regimens for medulloblastoma in children, and we discuss their contribution to improved survival and describe their toxicity profile and limitations.

## 1. Introduction

Medulloblastoma (MB) is the most common malignant brain tumor in childhood. Based on gene expression patterns in tumor tissue, MB can be classified into distinct subgroups. According to the current consensus, four main groups can be distinguished: wingless (WNT), sonic hedgehog (SHH), group 3 and group 4, with transcriptionally and genetically distinct profiles and correlated clinical outcomes [1]. The WNT subgroup MB has an excellent prognosis, whereas group 3 MB carries the worst prognosis [1,2,3]. The WHO consensus conference held in June 2015 recognized the importance of these biological groups, and the revised WHO classification of central nervous system (CNS) tumors published in 2016 defined MB both histologically and genetically [4]. The histologic classification consists of classic desmoplastic-nodular (DN), large-cell-anaplastic (LC/A) and MB with extensive nodularity (MBEN). The WHO genetic classification divides tumors into WNT-activated, SHH-activated-TP53 wild type, SHH-activated-TP53 mutant and non-WNT-SHH.

Using clinical risk stratification, MB in patients aged from 3 to 5 years up to 21 years at diagnosis with gross total or near total resection, with non-LC/A histological subtype and without *MYC* amplification has been considered a “standard-risk” (SR MB) disease, while the other patients are counted as “high-risk”, with infants and very young children posing therapeutic challenges.

Following the gold standard treatment based on surgery, reduced-dose craniospinal irradiation (CSI) and adjuvant chemotherapy, patients older than 3 years of age with SR MB have an expected 5-year overall survival (OS) around 85% [5,6,7]. In contrast to SR MB, no gold standard treatment has been defined for high-risk MB (HR MB,) and only around half of these patients are cured after conventional doses of CSI (e.g., 35–36 Gy) and chemotherapy [8,9]. Long-term survival rates have increased from 20–40% to up 60–70% in the last decade by using different intensive treatment strategies including high-dose chemotherapy (HDC) or alternative radiation therapy fractionation.

Infant and young children with MB represent a very challenging population to treat, given their worse prognosis compared to older children and, most notably, the increased vulnerability of the immature brain to neurotoxic agents. Among them, radiotherapy administered to the developing brain has been associated with unacceptable detrimental neurocognitive deficits hampering the independence and quality of life of these young children. These significant side effects associated with CSI have opened the field to innovative strategies for young children aiming at improving outcome while delaying, decreasing or avoiding its use. HDC was introduced as an alternative to CSI in young children with embryonal tumors based on the premise of their observed chemosensitivity.

In this review, we present the most recent trials using HDC regimens for HR and/or young patients with MB, and we discuss their contribution to improved survival and describe their toxicity profile and limitations.

## 2. High-Dose Chemotherapy in Children (>3 Years of Age) Newly Diagnosed with HR MB

### 2.1. Completed and Ongoing Trials

In the US and Europe, trials based on HDC strategies were developed to improve the outcome of children with HR MB. Table 1 summarizes the different HDC trials for patients. Firstly, Strother et al., investigated the feasibility of intensive chemotherapy regimens after surgical resection and CSI in the front-line treatment of children with MB and primitive neuro-ectodermal tumors (PNET) [10]. In this pilot study, after surgical resection, 19 patients with HR MB were treated with Topotecan in a 6-week phase II window, followed by risk-adapted CSI (M0-M1, 36 Gy; M2/M3, 39.6 Gy) and three-dimensional conformal boost to tumor bed (total dose, 55.8 Gy) and, where appropriate, local sites of metastases (total dose, 50.5 Gy). After a 6-week rest period, patients began 4 cycles of HDC (Cyclophosphamide, Cisplatin, and Vincristine), each followed by stem cell rescue. The 2-year progression-free survival (PFS) was 73.7% ± 10.5% [10]. This pilot study was expanded and a 5-year event-free survival (EFS) of 70% (95% CI, 55–85) was reached by SJMB-96 trial (NCT 00003211) for 48 patients with HR MB [11]. The phase III SJMB03 trial (NCT 00085202) was initiated to assess the clinical utility of molecular features in a uniformly treated patient cohort. In this trial, eligibility criteria were similar to the SJMB-96 trial, and the treatment regimen was the same except for the open window of Topotecan. The SJMB03 trial enrolled the largest cohort of patients with HR MB (*n* = 103) compared to SJMB-96, and the 5-year PFS rate was 58.7% (95% CI, 49.8–69.1%) [12]. In this SJMB03 trial, metastatic status was consistently associated with an inferior outcome, and stratification by clinical risk within molecular subgroups demonstrated inferior outcomes for HR MB in each subgroups except WNT. Among HR MB, 5-year PFS rates were 100% for WNT subgroup, 25% (95% CI, 7.5–83.0) for SHH, 40.6% (95% CI, 36.7–61.8) for group 3 and 68.1% (95% CI, 56.3–82.3) for group 4 [12].

In a monocentric Italian study using alternative radiation fractionation, HDC was used for metastatic patients with incomplete response to the treatment. A total of 32 evaluable patients received post-operative chemotherapy (Methotrexate, Etoposide, Cyclophosphamide, Carboplatin) in a 2-month schedule, followed by hyperfractionated accelerated radiotherapy (HART) with a maximal dose to the neuraxis of 39 Gy (1.3 Gy/fraction, 2 fractions/day) and a posterior fossa boost up to 60 Gy (1.5 Gy/faction/day). Fourteen patients with less than complete remission before HART received 2 courses of high-dose Thiotepa (300 mg/m^2^/day for 3 days) with stem cell rescue, while those in complete remission were given maintenance chemotherapy with Vincristine and Lomustine. The 5 year EFS rate of the whole cohort was 70% (SE +/−8%) [13]. In this monocentric study, no molecular data were available.

Recently, based on a pilot study [14], the phase II trial PNET HR + 5 (NCT 00936156) assessed the efficacy of a strategy based on HDC followed by conventional CSI. Fifty-one patients with HR MB were treated with 2 courses of conventional chemotherapy (Etoposide, Carboplatin), 2 courses of high-dose Thiotepa (200 mg/m^2^/day for 3 days), followed by conventional CSI (36 Gy on neuraxis; 54 Gy on primitive tumor bed) and maintenance treatment based on Temozolomide. The 5-year PFS for this cohort was 76% (95% CI, 63–86) [15]. In this trial with molecularly characterized HR MB, the excellent prognosis of WNT HR MB was confirmed: all patients with HR WNT MB (*n* = 3) were alive in first complete remission at 5 years. The survival for group 4 MB followed that of WNT MB with a 5-year PFS rate of 100%. No event was described at 5 years for group 4 MB (all metastatic at diagnosis), suggesting a possible treatment effect where the intensity of such a strategy may overcome the negative effect of high-risk features. For group 3 and SHH MB, the 5-year PFS rates were 59.3% (95% CI, 34.8–79.9) and 71.4% (95% CI, 35.9–91.8), respectively [15].

**Table 1:** High-dose chemotherapy clinical trials for children (>3 years of age) with newly diagnosed HR MB.

Trials (Reference)	*n*.	HR MB Criteria	Chemotherapy Regimen	HDC Regimen	Radiation Therapy (Dose Gy)		5-Year EFS (95% CI)
					CSI	Tumor Bed	
SJMB-96 [11]	48	Metastatic Postoperative residu ≥ 1.5 cm^2^	Window of topotecan	4 cycles post-CSI: CPM (4 g/m^2^), CDDP (75 mg/m^2^) and VCR (two 1.5 mg/m^2^ doses)	36 for M0–1 39.6 for M2–3	55.8	70% (55–85)
SJMB03 [12]	103	Metastatic Postoperative residu ≥ 1.5 cm^2^		4 cycles post-CSI: CPM (4 g/m^2^), CDDP (75 mg/m^2^) and VCR (two 1.0 mg/m^2^ doses)	36 for M0–1 39.6 for M2–3	55.8	56.7% ± 4.9%
HART [13]	33	Metastatic	4 courses before CSI: MTX, VP16, CPM, CBP 6 cycles of lomustine-VCR after CSI for patients in CR before CSI	2 cycles post-CSI: Thiotepa 900 mg/m^2^ (only for patients not in CR before CSI)	39	60	70% ± 8%
PNET HR + 5 [15]	51	Metastatic Postoperative residu ≥ 1.5 cm^2^ LCA MB MB with MYC amplification	2 courses before CSI: VP16-CBP 6 cycles of TMZ after CSI	2 courses before CSI: Thiotepa 600 mg/m^2^	36	54	PFS: 76% (63–86)

Gy: Gray; CPM: cyclophosphamide; CDDP: cisplatin; VCR: vincristine; MTX: methotrexate; CBP: carboplatin; TMZ: temozolomide; CR: complete remission.

### 2.2. Future Directions

All the recently published clinical trials with molecularly characterized HR MB confirm the excellent survival of WNT patients, irrespective of the regimens used [12,15,16]. These concurring findings are now opening the door to the possibility of a careful de-escalation of therapy for selected HR WNT patients, and de-escalation strategy without HDC has already begun for patients aged between 3 and 16 years with metastatic WNT in 3 currently accruing clinical trials (SJMB12, NCT01878617; PNET5, NCT02066220; ACNS 1422, NCT02724579).

For non-WNT HR MB, given the relatively limited size of the different cohorts published, the survival figures associated with the different strategies need to be cautiously interpreted and to be confirmed in larger cohorts. Moreover, trials with molecularly characterized HR MB [12,15] demonstrate the limitations of solely defined clinical risk stratification and highlights the power and need of combined molecular and clinical risk stratification to improve MB therapy for all. The future European trial for HR MB for children above 3 years old (SIOP HR MB) will be conducted to compare the HART approach versus the PNET HR + 5 strategy versus conventional chemotherapy with conventional radiotherapy. This trial will also give the opportunity to assess the clinical relevance of specific molecular features in a uniformly treated patient cohort.

## 3. High-Dose Chemotherapy in Young Children with Newly Diagnosed MB

### 3.1. Completed and Ongoing Trials

The use of HDC strategies as a first line of treatment for CNS embryonal tumors has been more widely adopted in North America than in Europe. Following initial phases I and II in relapse settings [17,18], North American study groups have progressively invested in the development of clinical trials dedicated to young patients newly diagnosed with CNS embryonal tumors based on an HDC backbone with two main conditioning regimens. Table 2 summarizes the different HDC trials for young children with newly diagnosed MB.

The Headstart group [19,20,21] has built up their experience over 4 consecutive trials using a single consolidation cycle of high-dose Thiotepa (300 mg/m^2^/d × 3 days), Etoposide (250 mg/m^2^/d × 3 days), and Carboplatin (AUC of 7/d × 3 days) following an induction phase of 3 to 5 cycles of conventional chemotherapy, including high-dose methotrexate (HD-MTX).

Based on previous CCG trials [22], the Children Oncology Group has pursued a consolidation phase with 3 sequential cycles of high-dose Thiotepa (10 mg/kg/d ×2 days) and Carboplatin (17 mg/kg/d × 2 days) following the usual number of 3 cycles of induction.

Using this backbone of 3 cycles of induction (Vincristine, Cyclophosphamide, Etoposide, Cisplatin) followed by 3 cycles of high-dose Carboplatin and Thiotepa and stem cell rescue, the recently closed COG study ACNS0334 evaluated, in a randomized manner, the contribution of HD MTX during induction to response rate and EFS for young patients with HR MB or with PNET. In this trial, HR MB was defined by the presence of metastatic disease at diagnosis and/or incomplete resection. Patients with non-metastatic DN MB were excluded. Adjuvant irradiation following consolidation was left to the physician’s discretion. Preliminary results have been presented in abstract form. Nineteen patients in the arm with HD methotrexate, and 20 in the arm without HD methotrexate, were evaluable for response. Following consolidation with HDC, the response rate was significantly higher in the HD MTX arm compared to the one without (63% versus 30% *p* = 0.038). Similarly, the patients who received HD MTX had a better 2-year EFS than those who did not (68.2% ± 9.6% versus 45.8% ± 13.8%; *p* = 0.08). The 5-year EFS for HR MB patients in the ACNS0334 HD MTX arm was also better than the one reported in the literature for previous protocols such as CCG9921, COG 99703 and HeadStart I and II [19,22,23]. Furthermore, the molecular subtyping was available in 38 patients with HR MB and identified 11 SHH, 25 group 3 and 2 group 4. All patients with SHH MB were metastatic at diagnosis, and the 5-year OS for this subgroup was 100% in both arms of treatment. Of particular interest, the addition of HD MTX was significantly beneficial to the patients with group 3 MB who achieved a 5-year OS of 80% compared to 40% for the group 3 patients treated without HD MTX (*p* = 0.025). Importantly, only 28% of the survivors received adjuvant RT for residual disease post-consolidation [24,25].

Running in parallel, the protocol Headstart III was a prospective phase 3 trial using intensive induction followed by myeloablative chemotherapy for children <4 years old with newly diagnosed localized MB, and children <10 years of age presenting either with disseminated MB or with postoperative residual tumor. The induction phase, including 5 cycles combining Etoposide, Cyclophosphamide, Cisplatin, Vincristine, Methotrexate and Temozolomide, was followed by one cycle of consolidation with a high dose of Carboplatin-Thiotepa-Etoposide. Adjuvant radiotherapy was only to be administered in children older than 6 years at diagnosis, or in children younger than 6 years with residual tumors post-induction. From 2003 to 2009, 92 patients with MB who were under 10 years at diagnosis were enrolled, of which 90% were under the age of 6 years. Fifty-two patients had classical MB, 27 DN, and 13 LC/A MB subtype. Fifty of the 68 (73%) evaluable patients who underwent myeloablative consolidation achieved a complete response or continuous complete response. The 5-year EFS and OS (±SE) rates for the entire cohort were 46 ± 5% and 62 ± 5%, respectively, and 38 ± 5% and 41 ± 5% for patients who were <6 years of age at diagnosis. Survival significantly differed by histological subtypes. DN MB had an excellent survival outcome, with a 5-year EFS and OS rates of 89 ± 6% and 89 ± 6% as compared to 26 ± 6% and 53 ± 7% for the patients with classic MB, and 38 ± 13% and 46 ± 14% for LC/A MB, respectively [21]. Noteworthy in this experience, patients with metastatic DN MB still achieved very good survival with a 5-year EFS and OS of 82 ± 12% and 81 ± 12% respectively, further suggesting that the negative prognostic value of metastatic status can be overcome by HDC for DN MB. This trial did not have molecular characterization of the cohort embedded in the objectives.

The Pediatric Brain Tumor Consortium (PBTC) led a multicenter study for young children newly diagnosed with CNS embryonal tumors investigating the feasibility of adding Vorinostat and Isotretinoin to a HDC chemotherapy backbone regimen (PBTC026). These 2 agents were administered during 3 classic cycles of induction combining Cisplatin, Etoposide, Cyclophosphamide and Vincristine. The consolidation phase was similar to the one on ACNS 0334 or CCG 99703 [22,24] using 3 cycles of high-dose Carboplatin and Thiotepa. Patients with localized MB received focal radiation therapy following consolidation therapy, and the remaining patients could receive radiation at the discretion of the treating physician, but CSI was not allowed in the protocol. Vorinostat and isotretinoin were then given in the maintenance phase for 12 cycles. Preliminary results of this trial have been presented in abstract form. Seventy-seven percent of the patients completed 3 cycles of induction therapy within the pre-specified feasibility endpoint of 98 days. The 2-year PFS and OS for the 20 patients with MB were 68.2% (±12.8%) and 77.0% (±11.1%) [26].

The French society of pediatric oncology (SFCE) led a multicenter Phase I/II trial (HR MB-5) for young children under 5 years of age with newly diagnosed HR MB assessing the efficacy of a strategy based on HDC with the intent to avoid radiotherapy. Following surgery, all children received 2 cycles of Etoposide-Carboplatin. If partial or complete response was achieved after induction chemotherapy, children received 2 courses of high-dose Thiotepa (600 mg/m^2^) with stem cell rescue. Patients in complete response after these 2 cycles of HDC received an additional cycle of HDC with Cyclophosphamide and Busulfan (Phase I part). Patients who did not achieve a partial response after induction or complete remission after high-dose Thiotepa received 2 cycles of Temozolomide-Irinotecan, followed by age-adapted CSI and maintenance treatment. Preliminary results of this trial have been presented in abstract form. The 3-year OS for the 28 patients with HR MB was 71.3% [52.7–84.7]. Among 13 children treated without radiotherapy, 9 patients were alive in complete remission (median follow-up 5 years) [27].

**Table 2:** Recent high-dose chemotherapy clinical trials for young children with newly diagnosed MB.

Trials	*n*	Chemotherapy Regimen	HDC Regimen	Radiation Therapy (RT)	Outcome
HeadStart III [21]	92	Induction 3 to 5 cycles of CDDP, CPM, VCR, VP16, HD MTX	1 cycle Thiotepa-VP16-CB	For children > 6 years or children not in CR	DN MB: 5-year EFS: 89% (±6) Classic MB: 5-year EFS: 26% (±6) LCA: 5-year EFS:38% (±13)
PBTC-026 [26]	20	Induction 3 cycles CDDP, CPM, VCR, VP16 with Vorinostat and isotretinoin Maintenance: Vorinostat and isotretinoin	3 cycles CB-Thiotepa	Focal RT For M0 MB At physician discretion for other patients	2-year PFS: 68.2% (±12.8)
ACNS0334 [24,25]	39	Induction 3 cycles CDDP, CPM, VCR, VP16 Randomized ± HD MTX	3 cycles CB-Thiotepa	At physician discretion	5-year EFS with HD MTX: 68.2% (±9.6%) 5-year EFS without HD MTX: 45.8% (±13.8%)
HR MB-5 [27]	28	Induction 2 cycles VP16-CB +2 cycles TMZ-Irinotecan for patient with insufficient response Maintenance TMZ treatment after RT	2 cycles thiotepa and for patients in CR: 1 cycle CPM-Busilvex	Age adapted CSI for patient with insufficient response to chemotherapy	3-year EFS: 42.3% (25.9–60.6%) Premature study closure for excess of event

CB Carboplatin; CDDP: cisplatin; CPM: cyclophosphamide; CSI: craniospinal irradiation; HDC: High dose chemotherapy; HD MTX: high-dose methotrexate; LCA: Large cell anaplastic; DN MB nodular desmoplastic medulloblastoma; M0 MB: non-metastatic medulloblastoma; RT: radiotherapy; VCR: vincristine; VP16: Etoposide; TMZ: Temozolomide, CR: complete remission.

### 3.2. Future Directions

These recent trials of HDC in young children with CNS embryonal tumors provide further evidence of the benefit of such intensive strategies to avoid CSI and improve survival for MB. However, several questions still need to be investigated to better refine the indication of HDC for MB in this young population. Do all young children with MB require HDC? Does adding sequences of treatment or new agents to the HDC backbone improve survival and/or allow further avoidance of adjuvant RT? Can we decrease or mitigate the toxicity associated with intensive consolidation with HDC?

#### 3.2.1. Young Children with DN/MBEN and/or SHH MB

Given the high cure rate of DN/MBEN and/or SHH MB consistently reported with HDC, the possibility of a prudent treatment de-escalation can reasonably be considered. In light of the failure of 2 recent clinical trials using conventional chemotherapy for this subgroup of infant MB [28,29], the current recommendation in North America continues to rely on HDC. The ongoing Headstart IV trial run by the NEXT consortium comprises a low-risk MB arm including children younger than 6 years with SHH MB p53 negative, irrespective of their metastatic status or extent of resection. The plan of treatment for this low-risk group included only 3 cycles of induction followed by a single cycle of consolidation (Carboplatin-Eoposide-Thiotepa). The investigators hypothesized this reduction of therapy will not be associated with inferior outcome compared to historical controls from the Headstart II and III trials. This arm of the Headstart IV trial recently closed after reaching targeted patient accrual. Matured data should be available in the coming years (NCT02875314).

Taking into account the excellent outcome for DN/MBEN MB reported on the HIT SKK 2000 regimen using conventional chemotherapy and serial intraventricular injection of methotrexate (5-year EFS and OS rates of 90% + 7% and 100% + 0%, respectively) in [30] and the Headstart III trial [21], international collaboration between European groups and the Next Consortium has been initiated to compare, in a prospective and randomized manner, these 2 regimens in young patients with “low-risk MB” defined by wild-type SHH MB. The trial would be the first one to place as a primary objective the question of neurocognitive outcome of these patients for the 2 regimens, which will be a significant novelty in the field.

Discussions are also currently being held within the COG to tackle treatment reduction for low-risk MB, to minimize some of the acute toxicities of HDC regimens by possibly decreasing the number of cycles of sequential HDC (Carboplatin-Thiotepa) and possibly integrating otoprotective agent during Cisplatin exposure. Such an approach would address the very clinically relevant issue of hearing toxicity in young children. It would also decrease the overall acute toxicity associated with previous HDC backbones [22,31] but will not be able to prevent the expected infertility associated with high-dose Thiotepa. It is likely that once reported, the matured data from the low-risk MB arm from Headstart IV might further shape the COG approach to dose reduction strategy, in an effort to address complementary questions for this low-risk group.

#### 3.2.2. Young Children with Non-DN and/or Non-SHH MB

Young children with non-DN/SHH MB will belong, in a large majority, to group 3 MB (30–40%) or to a lesser extent to group 4 MB (10–20%), WNT MB being extremely rare in children under 5 years of age [27,30,31]. However, due to differences in patients cohorts and analytical methods used to subtype MB, there is still significant difficulty in separating group 3 and group 4 MB from each other routinely [32]. It is therefore unknown whether group 3 and group 4 patients would benefit from different strategies upfront. Furthermore, the interplay between molecular subgrouping, clinical risk factors or additional molecular markers such as *MYC* status, for instance, still need to be deciphered. In the currently recruiting Headstart IV trial, all non-SHH MB are treated in the high-risk arm and are randomized to receive one cycle of Carboplatin-Thiotepa-Etoposide or tandem consolidation with 3 cycles of high-dose Carbopatin-Thiotepa. The hypothesis is that the tandem 3 cycles of consolidation will be associated with a better outcome compared to single consolidation without significant additional morbidities. Accrual on this arm is still ongoing. Post hoc analysis by molecular subgroup and clinical risk factors (metastatic status, extent of resection) of this cohort will also be critical to further refine treatment risk stratification.

#### 3.2.3. Young Children with Very HR MB

The optimum treatment for a remaining group of patients with higher-risk disease still needs to be proposed and studied. Namely, the young patients with group 3 and group 4 MB with metastatic disease at presentation and/or evidence of MYC amplification and patients with P53-mutated SHH MB do not seem to have benefited as much as others from the most current HDC strategies. While the addition to current HDC backbones of other modalities, such as maintenance therapy, intrathecal therapy and/or the use of focal radiation may be tempting, one has to be very careful of the risk of generating more toxicity for limited to no benefit. Although the addition of the combination of Vorinostat and isotretinoin was proven feasible, the contribution of such agents to improved survival still needs to be evaluated prospectively for these very high-risk patients. Preclinical studies would be informative to assess these new agents in models recapitulating specific molecular subgroups and associated molecular alterations. There is also a growing interest for incorporating intrathecal therapy as CNS prophylaxis or to intensify the treatment of CNS dissemination, which likely warrant further investigation. However, there is very limited data currently available on their efficacy and on toxicity when combined with HDC regimens. In light of the concern raised around the possible worse neurocognitive outcome in young patients treated with serial intraventricular injections of Methotrexate and conventional chemotherapy reported in the HIT SKK [33], further studies could investigate the use of other drugs administered directly into the cerebrospinal fluid which were previously evaluated, such as Topotecan, Mafosfamide or liposomal Cytarabine [34,35,36].

Within the SIOP European brain tumor committee, discussions are also being held to address the poor outcome of high-risk MB, which could rely on the HDC strategy with a randomized allocation of 2 different HDC regimens followed by the use of adjuvant age-adapted CSI (18 Gy or 23.4 Gy) based on post-induction tumor response.

## 4. Toxicity Associated with High-Dose Chemotherapy Regimen in Children

The toxicity of HDC strategies is not trivial for children, especially for very young patients. The improvement of supportive care measures and the increased mastery of expected complications have contributed to a considerable decrease in the mortality directly related to intensification of HDC regimens. While the rate of toxic death reported in the Headstart I and II experiences for non-metastatic MB was 14% (3/21) [20], it was brought down to 2% (2/92) in the most recent Headstart III [21], with one death occurring in the induction phase and one in the consolidation phase. More recent trials are associated with lower toxicity-related mortality rates, ranging from 0 to 6% [15,24,25,26,31]. For instance, no toxic deaths were described in trials for older children with HR MB (PNET HR +5, SJMB-96, SJMB03, and HART) [11,12,13,15] while on the ACNS 0334 cohort of young children with HR MB, 2 toxic deaths (5%) were described.

Nevertheless, the acute morbidity mainly related to myelosuppression remains a significant concern. Dhall et al., reported on the Headstart III trial that the main grade 4/5 toxicities during induction were related to myelosuppression, electrolyte abnormalities, and infection, whereas during consolidation, myelosuppression was the most common grade 4/5 toxicity. The author also described less frequent but severe complications in the form of single events of reversible encephalopathy, veno-occlusive disease, gastrointestinal hemorrhage, pulmonary hemorrhage and multi-organ system failure [21].

### 4.1. Ototoxicity

A significant long-term toxicity particularly relevant in this young population is the negative impact of such regimens on hearing, a critical function for the acquisition of speech and academic outcomes [37]. The HDC strategy heavily relies on platinum salts with a high cumulative dose of cisplatinum during induction and a high dose of carboplatin during the consolidation phase. The rate of severe ototoxicity reported in the most recent trials varies widely, in large part due to the toxicity grading systems used. The ototoxicity profile in ACNS 0334 has not yet been described, but in the similar trial ACNS0333 for ATRT, which had one less cycle of cisplatinum but the same consolidation, only one case of grade 4 ototoxicity by CTCAE was reported out of 65 evaluable patients [31]. In Headstart III, 7 cases of grade 3 ototoxicity and no grade 4 were reported in the cohort of 92 patients with MB [21]. This documentation of ototoxicity contrasts with previous reports on cohorts of young children receiving HDC regimens. In the phase I/II CCG 99703, which constitutes the backbone of ACNS0334 and included a similar high cumulative dose of cisplatinum to the Headstart regimens, Cohen et al., reported grade 3 and 4 ototoxicity rates were respectively 6.7% and 12.7% during induction and consolidation phases by CTCAE grading, but further documented 29% of hearing loss > 20 db at 500 to 4000 Hz [22]. Furthermore, in a retrospective series of 53 young children treated as per this same 99703, using the Chang ototoxicity classification [38], 45.5% of the children had a hearing loss greater or equal to grade 2 b (>20 and <40 dB at any frequencies below 4 kHz), and 39.3% required hearing support, either hearing aids or Frequency Modulating systems [39]. This remarkable discrepancy highlights the major need to report ototoxicity in young children in a consistent and, more importantly, in a clinically relevant manner. The SIOP Boston ototoxicity scale was found to detect more ototoxicity, and significantly earlier, compared to other grading systems, and is now being adopted more widely. While HDC strategies aim to protect the neurocognitive outcomes of young children by avoiding cranial radiation, they have the potential to indirectly affect intellectual ability and social development by significantly impairing their hearing function [40,41,42]. The ototoxicity of HDC strategies should therefore be considered as an important matter, and strategies to minimize it, either by decreasing the cumulative dose of platinum salts or by introducing otoprotective agents, should be further investigated in this young group of patients [43,44].

### 4.2. Neurocognitive Outcome

Investigating the neurocognitive status of young children following radiation-sparing approaches continues to be a challenge, and prospective and longitudinal evaluations of survivors are still too few. Of the 107 survivors of the Headstart III trial, 43 (40%) underwent neurocognitive assessment during follow-up. Twenty-four patients (56%) had MB and 12 (28%) received radiation therapy. As a group, the Headstart III patients performed within average to low-average range across all tested variables and no significant difference were found by diagnosis (MB, PNET and others) [45]. Fay McClymont et al., also reported the neurocognitive profile of 24 survivors of MB treated with HDC according to the protocol 99703. On average, the children were assessed 3.5 years (SD = 1.8) post-diagnosis, and the average full-scale intelligence score (FSIQ) was 92, ranging from 56 to 119. The majority of these children (74%) were functioning within low-average to average range, although clinically significant deficits (<10th percentile) in at least one area of neuropsychological functioning were found in 25% of the children [46]. Levitch et al., recently provided the long-term neurocognitive evaluation of patients treated on the Headstart II protocol between 1997 and 2003 [47]. In this study, 18 patients completed a neurocognitive battery and parents completed psychological questionnaires at a mean of 104.7 months post-diagnosis (standard deviation (SD) = 33.1). The mean age of these patients at diagnosis, at baseline testing and at long-term follow-up were 35.7 months old (SD 22.6), 45.7 months old (SD = 22.5) and 122.8 months old (SD = 45), respectively. There was no significant change in FSIQ at long-term follow-up (FSIQ = 92.13; SD = 20.8) compared to baseline. All other tested neurocognitive domains also remained stable except for a significant change in internalizing behaviors, with greater internalizing symptoms reported by parents at long-term follow-up evaluation [48]. These recent studies further position HDC strategies as safer alternatives in young children to minimize treatment-related neurocognitive impairment. As the number of long-term survivors treated during infancy with HDC strategies continue to increase, prospective evaluation of their neurocognitive outcome should become a more prominent priority and be integrated as part of the primary objectives of future clinical trials.

### 4.3. Neurotoxicity

In older children treated with HDC and CSI, MRI abnormalities have been described. In the SJMB-96 trial, among 134 patients with MB or supratentorial PNET treated prospectively with CSI followed by HDC, 22 developed white matter lesions at a median of 7.8 months after starting therapy (range, 1.9–13 months). Lesions were predominantly in the pons and cerebellum. Sixteen patients had MRI-abnormality resolution at a median of 6.2 months (range, 1.68–23.5 months) after onset. Two patients developed necrosis and atrophy. Patients with white matter lesions had significant declines in estimated IQ (−2.5 per year) [47]. In a HART study, 6 of 14 patients treated with high-dose Thiotepa after radiation therapy had therapy-induced lesions on MRI, and half of the children had neurologic or symptoms judged to be related to radiation-induced changes [13]. Thust et al., reported brain MRI abnormalities in 11 of 14 children receiving dose-intensive sequential, HART, and Thiotepa [49]. Ruben et al., found that chemotherapy after radiotherapy led to an approximately 5-fold increase in the risk of cerebral necrosis [50]. In the HART study and SJMB-96 trial, patients received radiotherapy followed by HDC.

### 4.4. Gonadal Dysfunction

Pediatric patients with brain tumors are at high risk for treatment-related hormone deficiencies. The risk of primary gonadal dysfunction is more frequent among female survivors. Sterilization and loss of hormone production following treatment with alkylating agents and radiation therapy are usually concurrent in females because hormone production is closely linked to the presence of ova and maturation of the primary follicle [51]. De Wire et al., estimated the cumulative incidence of primary ovarian insufficiency in 30 female patients with newly diagnosed embryonal CNS tumors treated with risk-adapted CSI followed by HDC with stem cell support (SJMB-96 trial). In this study, with a median length of follow-up of 7.1 years (range, 4.0–10.8 years), the cumulative incidence of primary ovarian insufficiency 6 years after completion of radiation therapy was 82.8% [52]. The median estimated radiation doses were 5.6 Gy (range, 0.7–30.5 Gy) and 6.1 Gy (range, 0.6–31.9 Gy) to the right and left ovaries, respectively. The incidence of ovarian dysfunction was similar among patients who received ovarian radiation therapy < 5 Gy compared to those who received ≥ 5 Gy [53], similar to other previous reports [53,54], suggesting that ovarian insufficiency was more likely due to alkylating agent. The doses of alkylating agents such as Melphalan, Busulfan or Thiotepa used during consolidation are highly likely to be associated with significant risk of future infertility or premature ovarian failure in young women, not only because of the high cyclophosphamide equivalent dose administered, but also because of the prepubertal status of these children [55,56]. Although the older survivors of HDC strategies for MB are now in their 30’s, even retrospective studies investigating gonadal function and fertility status in this population has yet to be undertaken.

### 4.5. Nephrotoxicity

Among other toxicities less studied in this population, Elborai et al., recently described glomerular filtration alterations and nephrotoxicity observed in a cohort of young children with MB and ATRT who received high-dose carboplatin as part of their consolidation. Nephrotoxicity severity ranged from acute transient to post-transplantation renal failure requiring dialysis [57]. This limited study called for close monitoring of renal function and more scrutinized long-term evaluation in the survivors’ population.

### 4.6. Second Malignancies

Although the avoidance of radiation diminishes the risk of treatment-induced second malignancies, alkylating agents and inhibitors of topo-isomerase carry by themselves the potential of inducing second cancers such as acute leukemia. To date, none of the Headstart or COG/CCG reports have mentioned the occurrence of second malignancies, with some of those patients being treated in the late 1990s. In the PNET HR + 5 trial, 2 patients (3.9%) developed secondary tumors, one in the context of Li-Fraumeni syndrome [15]. This rate of second malignancies appears aligned with frequencies reported from protocols based on adjuvant conventional chemotherapy and CSI [58,59,60]. The long-term follow-up study of Children’s Oncology Group trial A9961 estimated a cumulative 10-year incidence rate of secondary malignancies of 4.2% (95% CI, 1.9% to 6.5%) [58]. Among 280 patients with MB in the HIT91 trial, a second tumor occurred in 12 (4.3%) cases [61]. The 10-year cumulative incidence rate of second malignancies among MB survivors of multimodal therapy at St. Jude Children’s Research Hospital was 5.5% (95% CI, 2.8% to 9.6%) [60]. The increased cumulative incidence of second malignancies after multimodal therapy may be partially attributable to the improvement in neuro-imaging techniques to detect indolent tumors. The etiology of subsequent neoplasms is multifactorial and includes primary cancer therapy as well as genetic susceptibility. Retrospective collaborative studies will help delineate the contributing role of HDC, adjuvant radiation and possibly underlying genetic predisposition such as SUFU germline mutation in survivors of SHH MB [62].

## 5. Conclusions

The recent trials of HDC have led to improved survival in HR MB and decreased treatment-related toxicity in young children with MB. Ongoing and upcoming clinical trials based on more refined risk stratification integrating molecular subgrouping should help address whether HDC strategies are needed for all young children with MB and explore careful treatment de-escalation for some of them. For the remaining HR MB patients with currently unsatisfactory survival, building up on previous HDC backbones by adding new agents or other modalities of treatment seems to be the current direction considered. Greater vigilance is required with regard to the risk of additional or worse toxicity that could be induced with more treatment.
